# Acute Exercise Fatigue Impairs Cognitive Control: Neurophysiological Mechanisms Revealed by ERP and ERSP Analyses

**DOI:** 10.3390/biology14121688

**Published:** 2025-11-27

**Authors:** Shuqi Yao, Hongwei Lu, Longhai Zhang, Fujie Liu, Fuhai Ma, Aiping Chi

**Affiliations:** 1 School of Physical Education, Shaanxi Normal University, Xi’an 710062, China; 2 School of Physical Education, Qinghai Minzu University, Xining 810007, China

**Keywords:** cognitive control, exercise-induced fatigue, P3 component, N2 component, event-related potentials, event-related spectral perturbations

## Abstract

This study aimed to investigate the effects of acute exhaustion exercise on cognitive control in young men, a key higher cognitive function for goal-directed behavior. Although long-term regular exercise benefits cognition, the effects of acute exhaustion exercise on cognitive control and its neural mechanisms are not fully understood. 35 male college students completed a Stroop task before and after exhaustion exercise on a cycle ergometer with incremental load. Electroencephalogram data were collected synchronously during the task. Behavioral measures (accuracy, reaction time), Event-Related Potential components (N2, P3 amplitudes and latencies), and Event-Related Spectral Perturbation (energy changes in theta, alpha, beta frequency bands) were analyzed. Results: Behavioral results showed that task accuracy only significantly decreased under the conflict condition (incongruent trials) following exhaustive exercise. ERP analysis revealed that the P3 amplitude at the anterior site (Fz) was significantly reduced post-exercise, but specifically for the incongruent condition, while the N2 amplitude demonstrated a more widespread enhancement. Time–frequency analysis found a significant decrease in alpha-band power over the parietal region after exercise. Theta and beta band activities were not significantly affected by exercise-induced fatigue. Conclusions: Acute exhaustive exercise did not impair early conflict monitoring functions (as indicated by stable N2 component and theta oscillations), but it compromised later higher-order cognitive processes related to attentional resource allocation and conflict resolution (manifested as reduced anterior P3 amplitude), accompanied by decreased efficiency in neural oscillatory activity associated with inhibitory control (reduced alpha power). This suggests that fatigue primarily affects the neural mechanisms of the “implementation” stage rather than the “monitoring” stage in the cognitive control cascade.

## Simple Summary

We all know that intense physical exercise can make us feel tired, but how does this exhaustion affect our ability to think clearly? This study investigated how acute, exhaustive exercise impacts a specific mental skill called “cognitive control”—the ability to override automatic impulses and focus on a goal. We had young male participants perform a color-word matching task (the Stroop task) before and after cycling to exhaustion. We measured their brain activity using electroencephalography (EEG). The results showed that after exhaustive exercise, participants made more errors, but only during the more difficult trials that required them to inhibit a strong automatic response. Brain activity analysis revealed that the initial brain signal for detecting a mental conflict remained intact. However, the later brain processes responsible for resolving that conflict and allocating mental resources were significantly weakened. In essence, when you are physically exhausted, your brain can still identify a problem, but it struggles to effectively implement the solution. These findings are crucial for understanding performance in high-stakes professions like athletics, military operations, and emergency services, where critical decisions must be made under extreme physical fatigue.

## 1. Introduction

Cognitive control, also known as executive function, refers to a set of higher-level cognitive processes that enable individuals to adapt their thoughts and behaviors to changing situations and task requirements. It is essential for tasks such as planning, decision-making, problem-solving, and inhibiting inappropriate responses [1,2]. Cognitive control is primarily regulated by the prefrontal cortex and its interconnected neural networks [3]. The Stroop task is commonly used to assess cognitive control as it effectively creates response conflict and inhibition demands [4]. In this task, individuals must ignore word meanings and instead focus on reporting the color of the text, requiring strong inhibitory control to override the automatic reading response.

The relationship between exercise and cognitive function is complex and multidimensional [5,6]. While regular physical exercise has been shown to improve cognitive function, acute and high-intensity exercise leading to fatigue may have temporary negative effects on cognitive performance. Exercise-induced fatigue refers to the inability of organic physiological processes to sustain their functions at a specific level and/or to maintain a predetermined exercise intensity [7]. It is a complex physiological and psychological state involving changes in multiple central and peripheral systems [8]. Research has shown that both physical and mental fatigue can lead to a decline in cognitive functions such as decision-making, attention, and response inhibition [9]. This phenomenon can be explained by the cognitive resource theory, where high-intensity physical exertion competes for limited brain resources with cognitive tasks, resulting in impaired cognitive performance [10]. However, the specific mechanisms of how acute exhaustive exercise affects different neurocognitive processing stages are not fully understood.

The event-related potential (ERP) technique provides a powerful tool for investigating the dynamic neural activity underlying cognitive processes with its millisecond-level time resolution. The Stroop paradigm, as a classic conflict monitoring task, effectively activates the neural circuitry of the dorsolateral prefrontal cortex and anterior cingulate cortex (ACC) [11,12,13]. Two key ERP components in the Stroop task-N2 and P3, are closely related to cognitive control. The N2 component, primarily distributed in the frontal-central region, is considered a neural index of conflict monitoring, reflecting the brain’s early detection of inconsistent information in tasks [14]. The P3 component, mainly distributed in the parietal region, is associated with late cognitive processes such as attention resource allocation, stimulus evaluation, and working memory updating. Existing studies generally suggest that physical exercise can significantly optimize neural resource allocation related to selective attention, manifested as an enhanced P3 component amplitude or a shortened latency [11,15,16,17,18]. Chu et al. suggested that adolescents show improved inhibitory control after acute exercise, accompanied by an enhanced P3 component [19]. Aly and Kojima’s randomized controlled trial confirmed that moderate-intensity exercise can enhance neural resources related to perception and cognition [20]. However, these studies mainly focus on moderate-intensity exercise, with less exploration of the effects of incremental exercise to exhaustion on cognitive function.

In addition to ERP analysis in the time domain, time–frequency analysis such as event-related spectral perturbation (ERSP) can reveal task-related neural oscillatory changes, providing richer information. Different frequency bands of neural oscillations are associated with specific cognitive functions: enhanced theta band (4–7 Hz) activity is typically related to cognitive control and conflict resolution mediated by the frontal lobe [21]; changes in alpha band (8–13 Hz) energy, particularly its suppression (increase in energy), is considered a sign of active suppression of irrelevant information or regions [22]; while beta band (14–30 Hz) activity is associated with motor preparation and response execution [23].

Although prior research has explored the impact of acute high-intensity exercise on cognitive performance, the neurophysiological mechanisms—particularly in the domain of neural oscillations—remain poorly understood. Most studies have relied solely on behavioral measures or ERP components, leaving a gap in how exercise-induced fatigue modulates oscillatory brain activity during cognitive control tasks. The present study addresses this gap by integrating ERP and ERSP analyses to provide a multi-dimensional assessment of the neural dynamics underlying impaired cognitive control following acute exhaustive exercise.

We make the following hypotheses: (1) Acute exhaustion exercise will impair the behavioral performance of the Stroop task, especially under high-conflict conditions. (2) This behavioral impairment will be accompanied by changes in neurophysiological indicators, specifically manifested as prolonged P3 latency (slower stimulus evaluation) and compensatory or deteriorating changes in P3 amplitude. (3) In the time–frequency domain, we expect fatigue to interfere with neural oscillations related to cognitive control, possibly manifested as a decrease in theta band energy and/or a decrease in alpha band suppression ability. Through this multidimensional analysis, we hope to more comprehensively reveal the neural mechanisms underlying the impact of acute exercise fatigue on cognitive control.

## 2. Materials and Methods

### 2.1. Participant

The study conducted a priori power analysis using G*Power 3.1 software. The analysis was set as follows: effect size f = 0.25, α level of 0.05, statistical power of 0.80, and within-subject correlation of 0.5 [24,25]. The calculation showed that 28 participants were needed to achieve sufficient statistical power. Considering potential dropouts, a total of 35 healthy male university students were recruited for the experiment. All participants met the following criteria: (1) good health with no history of genetic diseases, brain injuries, cardiovascular diseases, mental or neurological disorders; (2) normal uncorrected or corrected vision with no color blindness; (3) no recent physical injuries or excessive fatigue; (4) right-handed; (5) regularly engaged in physical activity (International Physical Activity Questionnaire IPAQ); (6) pre-experiment requirements: avoid intense exercise, alcohol, caffeine, and medication intake within 24 h before testing, maintain emotional stability; (7) informed consent: all participants fully understood the experimental procedures, voluntarily signed informed consent forms, and were compensated after the experiment. The basic information about the participants is shown in Table 1.

### 2.2. Establishment of the Exhaustion Model

The Ergoline 800 power bike (Bitz, Germany) was used as the experimental equipment, equipped with an electronic resistance adjustment system that allows researchers to set specific power outputs for incremental load to exhaustion exercise. Participants performed a one-time exhaustion exercise using an incremental load protocol. They cycled on the power bike with increasing loads, starting at 50 watts and increasing by 50 watts every 3 min until exhaustion. The fourth level did not increase the load or limit the time, continuing until exhaustion. Before the formal test, participants completed a 3 min warm-up at 25 W. During the exhaustion exercise, heart rate changes were monitored using a physical activity monitor (GT9-X, ActiGraph, Pensacola, FL, USA) worn on the participant’s left hand. Additionally, at the end of each level, participants were asked to rate their perceived exertion using the Rating of Perceived Exertion (RPE) scale.

The criteria for terminating the exercise due to exhaustion are when the participant experiences three of the following four conditions [26]: (1) physical condition: difficulty breathing and profuse sweating; (2) blood pressure changes: systolic pressure > 150 mmHg, diastolic pressure > 75 mmHg; (3) heart rate changes: participant’s heart rate reaches or approaches their maximum heart rate (220-age); (4) RPE level: participant’s RPE level reaches 18 or higher, and after multiple encouragements, they still choose to discontinue the exercise.

### 2.3. Cognitive Task Assessment

**Figure 1:**
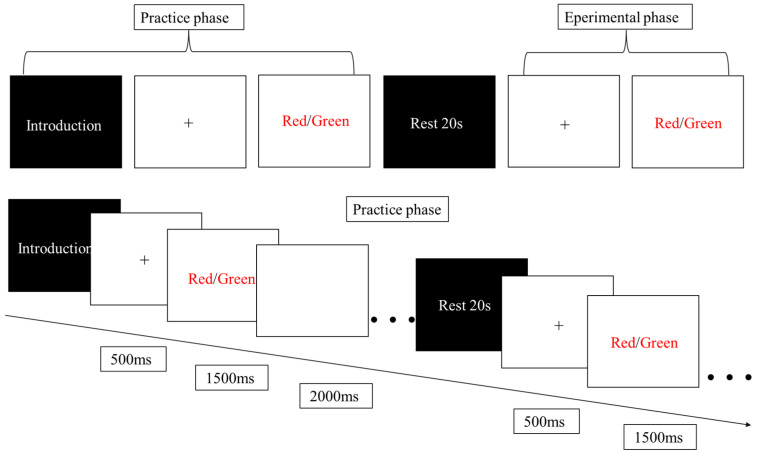
Stroop paradigm task flowchart.

Use E-prime 3.0 (Psychology Software Tools, Pittsburgh, PA, USA) to create a Stroop task stimulus program with 4 blocks of stimuli (2 congruent, 2 incongruent). The stimuli consist of the words “red,” “blue,” “green,” and “yellow,” presented in random order in red, blue, green, and yellow colors. In the congruent condition block, the stimuli are words that match the color they are presented in (e.g., the word “red” in red font); in the incongruent condition block, the stimuli are words that do not match the color they are presented in (e.g., the word “green” in red font). All stimuli are presented in the center of the screen against a black background. Participants are instructed to respond based on the actual color of the Chinese characters, not their meaning, using the “F” key for red (left middle finger), “G” key for blue (left index finger), “H” key for green (right index finger), and “J” key for yellow (right middle finger). Each trial consists of: (1) a 500 ms white fixation cross “+”; (2) a 1500 ms presentation of the Chinese character stimulus; (3) a 2000 ms blank screen interval. Each block includes 15 randomly presented trials, with congruent and incongruent conditions randomly intermixed lasting 60 s. There is a 20 s break between blocks, with 20 s rest periods before and after the experiment. A practice session is included before the actual experiment to ensure participants understand the task requirements. See Figure 1 for a schematic representation of the experimental procedure.

### 2.4. EEG Data Acquisition and Processing

Electroencephalogram (EEG) data were collected using the 32-channel EEG signal acquisition system from Neuroscan company (Neuroscan, Charlotte, NC, USA). The electrode cap was installed according to the international 10–20 system standard. During EEG testing, the laboratory environment was kept quiet with appropriate lighting and temperature. Prior to testing, participants were required to wash their scalp to remove oil and ensure all electrode impedances were adjusted to below 5 kΩ. Impedances were rechecked after exhaustive exercise to ensure signal quality. Participants performed the Stroop task paradigm before and immediately after exhaustive exercise, while EEG data were simultaneously recorded. The EEG signal was sampled at a rate of 1000 Hz, with bandpass filtering from 0.05 to 100 Hz and notch filtering at 50 Hz. The reference electrodes used were located at the bilateral mastoids (M1/M2).

We utilized the EEGLAB toolbox within MATLAB (R2022b; MathWorks, Natick, MA, USA) for data preprocessing. This involves implementing bandpass filtering (0.5~100 Hz) and notch filtering (48~52 Hz), reducing the sampling rate to 500 Hz, using the bilateral mastoids (M1, M2) as reference electrodes, eliminating time segments with substantial drift, and documenting faulty electrodes. We used the Independent Component Analysis (ICA) algorithm to rectify potential artifacts such as eye movement and other unwanted signals such as muscle and heart activity. We scrutinized and confirmed the attributes of individual independent components.

Prior to statistical analysis, we removed trials with behavioral response errors and applied rigorous artifact rejection to the EEG data (rejection criteria: voltage changes exceeding ±100 μV). The mean behavioral accuracy across participants was 97.42% (SD = 1.52%), indicating a very low proportion of trials (2.58%) were excluded due to performance errors. After the complete preprocessing pipeline, an average of 29.3 valid trials per condition per participant (SD = 0.15) were retained for final analysis. Paired sample t-tests confirmed that the number of valid trials did not differ significantly between conditions (congruent vs. incongruent: t(34) = 0.45, *p* = 0.653) or sessions (pre- vs. post-exercise: t(34) = 1.77, *p* = 0.086), ensuring no systematic bias was introduced by the trial exclusion process.

### 2.5. ERP Time-Domain Analysis

The P3 component is closely related to advanced cognitive functions such as working memory updating and context information integration [27,28], and its amplitude and latency changes can effectively reflect the dynamic regulation of cognitive resource allocation [29]. The N2 component, as an important indicator reflecting conflict monitoring and cognitive control, is closely related to executive functions [30,31]. This study focuses on the characteristics of N2 and P3 components induced by the Stroop task. Specifically, the analysis window is set from −500 ms~2500 ms relative to the stimulus presentation time, with baseline correction using the average amplitude from −500 ms~0 ms. The specific time windows for the N2 and P3 components are determined based on the total average waveforms, and the following measures are extracted under both congruent and incongruent conditions: (1) the mean amplitude value (mean within the peak ± 10 ms time window [32,33]); (2) the peak latency (time interval from stimulus presentation to peak). Based on previous research evidence and pilot study results, we focus on the frontal (Fz), fronto-central (FCz), central (Cz), central-parietal (CPz), and parietal (Pz) regions of the brain [34,35,36,37]. The N2 component is identified in the window of 200–300 ms post-stimulus [38,39], while the P3 component shows a clear peak around 300–400 ms post-stimulus [40].

### 2.6. Time–Frequency Analysis

This study utilized Short-Time Fourier Transform to conduct time–frequency analysis on each trial and calculate ERSP. Based on previous research evidence, we focused on the neural oscillatory characteristics in the theta (4~7 Hz), alpha (8~13 Hz), and beta (14~20 Hz) frequency bands. The analysis parameters for each frequency band were set as follows: For the theta band analysis, a time window of 200~500 ms was selected, corresponding to the conflict monitoring stage associated with the N2 component [41]. Electrode selection focused on the ACC area (FCz, Cz), and the frontal lobe (Fz) cortex, regions known to be closely related to conflict monitoring function [42,43,44].For the alpha band analysis, a time window of 300~550 ms was set, covering the inhibition control process related to the P3 component [45]. Analysis primarily targeted the midline parietal region (Cz, Pz, CPz), known to reliably reflect alpha rhythm activity associated with inhibition control [46]. In the beta band analysis, we specifically examined the activity of low-frequency beta within the 250~450 ms time window, corresponding to the motor preparation stage [47,48]. Electrode selection focused on the central motor area (FCz, Cz), typical representation sites for neural activity related to motor preparation [49,50].

### 2.7. Statistical Analysis

All data in this study were analyzed using SPSS 27 software. For ANOVA results, mean ± standard deviation (Mean ± SD) values were used for representation, while median and quartiles were used for non-parametric test results. The Kolmogorov–Smirnov test was conducted to check for normal distribution of data. If the data did not follow a normal distribution, the Friedman test and Mann–Whitney U test were used to compare differences between groups. Repeated measures ANOVA was used for normally distributed data with factors of consistency (consistent vs. inconsistent) and time (before exercise vs. after exercise). For the ERP and ERSP data, we employed a three-way repeated-measures ANOVA with factors including Time (pre-exercise, post-exercise), Congruency (congruent, incongruent), and Electrode. Mauchly’s sphericity test was used to check for the sphericity assumption in repeated measures ANOVA. The Greenhouse-Geisser correction was applied if the sphericity assumption was violated, and the Bonferroni correction was used for post hoc pairwise comparisons. Effect sizes in ANOVA were reported using partial eta-squared (η^2^), with values > 0.05 indicating a small effect, >0.10 indicating a medium effect, and >0.20 indicating a large effect. Correlation analysis was conducted to examine the relationship between changes in ERP and ERSP components before and after exhaustive exercise with changes in behavioral indicators. Pearson correlation analysis was used to assess the relationship between behavioral and ERP components. Two-tailed tests were used for all analyses, and the False Discovery Rate correction was applied for multiple comparisons. Significance levels were set at *p* < 0.05 for significance and *p* < 0.01 for high significance.

## 3. Results

### 3.1. Evaluation of the Exhaustion Model

All 35 participants successfully completed the incremental exhaustion exercise program without any dropouts or adverse reactions. The evaluation indicators of the exhaustion exercise model are shown in Table 2 (Table S1), indicating that the participants reached a state of physiological exhaustion at the end of the exercise. (1) At the termination of the exercise, the participants’ average maximum heart rate reached 183.94 ± 9.37 beats/min. Calculated based on the estimated maximum heart rate for their age (220-age) of approximately 196.5 beats/min, the participants on average achieved 93.6% of their personal predicted maximum heart rate, meeting the heart rate criteria for exhaustion exercise. (2) Subjective fatigue perception: The participants reported a high level of subjective fatigue perception at 19.14 ± 0.36, close to the maximum value on the scale (20), indicating an “extreme fatigue” level, suggesting that the participants subjectively could not maintain the current exercise intensity or found it extremely difficult. (3) Duration of exercise: The average time for participants to reach exhaustion from the start of the exercise was 13.95 ± 3.20 min, falling within the typical range for exhaustion induced by the incremental load protocol, reflecting that the protocol provided a sufficient exercise load.

In conclusion, based on multiple dimensions such as heart rate, RPE, and physiological performance, the incremental load power cycling protocol used in this study can effectively and safely induce subjects to reach exhaustion, successfully establishing an exhaustion exercise model. This provides a reliable physiological basis for future research on the impact of exhaustion exercise on cognitive function.

### 3.2. Behavioral Results

Accuracy: The results showed a significant main effect of time (F(1, 34) = 5.858, *p* < 0.05, η^2^ = 0.146). Simple effect analysis revealed a significant decrease in accuracy after exercise in the incongruent condition (*p* < 0.05). There was also a significant main effect of congruency (F(1, 34) = 10.864, *p* < 0.05, η^2^ = 0.175), with accuracy significantly lower in the incongruent condition compared to the congruent condition after exercise (*p* < 0.05). The interaction between time and congruency was not significant (*p* > 0.05). Reaction time: A two-way repeated measures ANOVA showed that the main effects of time, congruency, and the interaction between time and congruency were not significant (*p* > 0.05) Table 2 (Table S1).

### 3.3. ERP Time-Domain Results

This study employed a 2 (congruency: congruent vs. incongruent) × 2 (time: pre-exercise vs. post-exercise) × 5 (electrode: Fz, FCz, Cz, CPz, Pz) three-way repeated-measures ANOVA to examine changes in the mean amplitude and latency of the P3 and N2 components. The P3 and N2 mean amplitudes and latencies for each electrode are detailed in Appendix A and Appendix B.

#### 3.3.1. P3 Mean Amplitude

A three-way repeated-measures ANOVA with factors Time (pre-movement, post-movement), Congruency (congruent, incongruent), and Electrode site (Fz, FCz, Cz, CPz, Pz) was performed on P3 amplitude. The results (Table 3 and Table S3) revealed a significant main effect of Congruency (F(1, 34) = 4.581, *p* = 0.040, η^2^ = 0.119), with smaller P3 amplitudes in the incongruent condition compared to the congruent condition. A significant main effect of Electrode site was also observed (F(1.688, 57.386) = 12.107, *p* < 0.001, η^2^= 0.263). Post hoc comparisons indicated a posterior-to-anterior increasing gradient of P3 amplitude, with significantly larger amplitudes at parietal sites (Pz, CPz) than at anterior sites (Fz, FCz). The main effect of Time was not significant (F(1, 34) = 1.287, *p* = 0.265, η^2^ = 0.036). Critically, a significant three-way interaction of Time × Congruency × Electrode site was found (F(1.800, 61.195) = 7.307, *p* = 0.002, η^2^ = 0.177).

**Figure 2:**
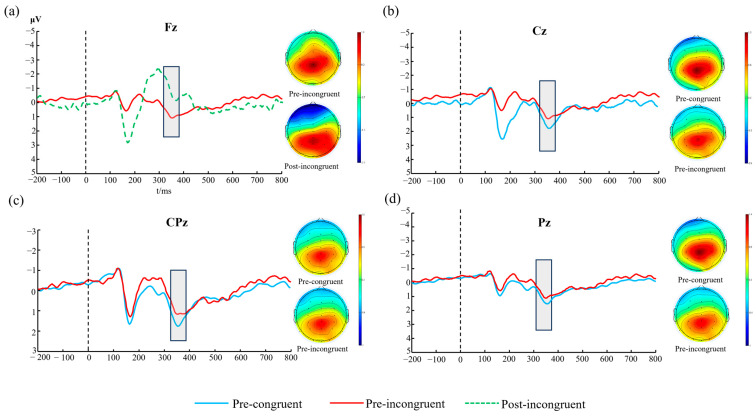
P3 Mean Amplitude Across Electrodes and Conditions. Note: (**a**) P3 amplitude at Fz electrode; (**b**) P3 amplitude at Cz electrode; (**c**) P3 amplitude at CPz electrode; (**d**) P3 amplitude at Pz electrode.

To decompose this three-way interaction, separate two-way repeated-measures ANOVAs (Time × Congruency) were conducted for each of the five electrode sites. At the Fz site, a significant main effect of Time was observed (F(1, 34) = 5.979, *p* = 0.020, η^2^ = 0.150). Simple effect analysis further revealed that P3 amplitude significantly decreased after movement under the incongruent condition (*p* = 0.004; Figure 2a). Neither the main effect of Congruency nor the Time × Congruency interaction reached significance (*p* > 0.05). At the FCz site, a significant main effect of Congruency was found (F(1, 34) = 5.173, *p* = 0.029, η^2^ = 0.132), reflecting significantly reduced P3 amplitudes in the incongruent condition (*p* < 0.05). The main effect of Time and the Time × Congruency interaction were not significant (*p* > 0.05).At the Cz site, the main effect of Congruency was significant (F(1, 34) = 5.753, *p* = 0.022, η^2^ = 0.145). Simple effect analysis indicated that P3 amplitude was significantly lower in the incongruent condition than in the congruent condition before movement (*p* = 0.005; Figure 2b). The main effect of Time and the interaction were not significant (*p* > 0.05).At the CPz site, the main effects of Time and Congruency, as well as their interaction, were not significant (*p* > 0.05). However, simple effect analysis showed that before movement, P3 amplitude was significantly lower in the incongruent condition compared to the congruent condition (*p* = 0.024; Figure 2c).At the Pz site, a significant Time × Congruency interaction was identified (F(1, 34) = 4.770, *p* = 0.036, η^2^ = 0.123). Subsequent simple effect analysis demonstrated that after movement, P3 amplitude was significantly lower in the incongruent condition relative to the congruent condition (*p* = 0.021; Figure 2d).

#### 3.3.2. P3 Peak Latency

A three-way repeated-measures ANOVA with factors of Time (pre-exercise, post-exercise), Congruency (congruent, incongruent), and Electrode (Fz, FCz, Cz, CPz, Pz) was conducted on P3 latency. The results revealed a significant main effect of Electrode (F(2.91, 98.92) = 13.07, *p* < 0.001, η^2^ = 0.278), indicating significant differences in P3 latency across electrode locations. The Time × Electrode interaction was also significant (F(2.66, 90.30) = 5.21, *p* = 0.003, η^2^ = 0.133). Furthermore, the three-way Time × Congruency × Electrode interaction reached statistical significance (F(2.68, 91.24) = 13.17, *p* < 0.001, η^2^ = 0.279).

Based on the significant three-way interaction, simple effect analyses of Time × Congruency were subsequently performed at each electrode site. The results indicated a significant main effect of Time only at the Pz electrode site (F(1, 34) = 9.01, *p* = 0.005, η^2^ = 0.209). Further simple effect analysis revealed that under the congruent condition, P3 latency was significantly shorter post-exercise compared to pre-exercise (*p* = 0.019). No significant main effects of Time or Congruency, nor Time × Congruency interactions, were observed at other electrode sites (*p* > 0.05).

#### 3.3.3. N2 Mean Amplitude

The three-way repeated-measures ANOVA revealed significant main effects of Time [F(1, 34) = 9.933, *p* = 0.003, η^2^ = 0.226] and Congruency [F(1, 34) = 5.989, *p* = 0.020, η^2^ = 0.150], while neither the main effect of Electrode nor any interaction effects reached statistical significance (Table 3 and Table S3). Post hoc analysis of the significant Time main effect indicated that the N2 mean amplitude was significantly more negative post-exercise compared to pre-exercise, suggesting an enhanced negative deflection of the N2 component following exhaustive exercise. For the significant Congruency main effect, the incongruent condition elicited significantly more negative N2 amplitudes than the congruent condition, demonstrating the typical conflict effect in the Stroop task.

#### 3.3.4. N2 Peak Latency

The three-way repeated-measures ANOVA revealed significant main effects of Electrode [F(2.374, 80.728) = 20.054, *p* < 0.001, η^2^ = 0.371] and significant Time × Electrode [F(3.285, 111.704) = 6.299, *p* < 0.001, η^2^ = 0.156] and Time × Congruency × Electrode [F(2.622, 89.156) = 16.872, *p* < 0.001, η^2^ = 0.332] interaction effects (Table 3). Given the significant three-way interaction, separate 2 (Time) × 2 (Congruency) repeated-measures ANOVAs were conducted at each electrode site. The results revealed a significant main effect of Congruency at the Cz electrode site [F(1, 34) = 8.419, *p* = 0.006, η^2^ = 0.198], indicating a significant difference in N2 latency between the congruent and incongruent color-word conditions.

### 3.4. Event-Related Spectral Perturbation Results

#### 3.4.1. Theta Band Analysis Results

**Figure 3:**
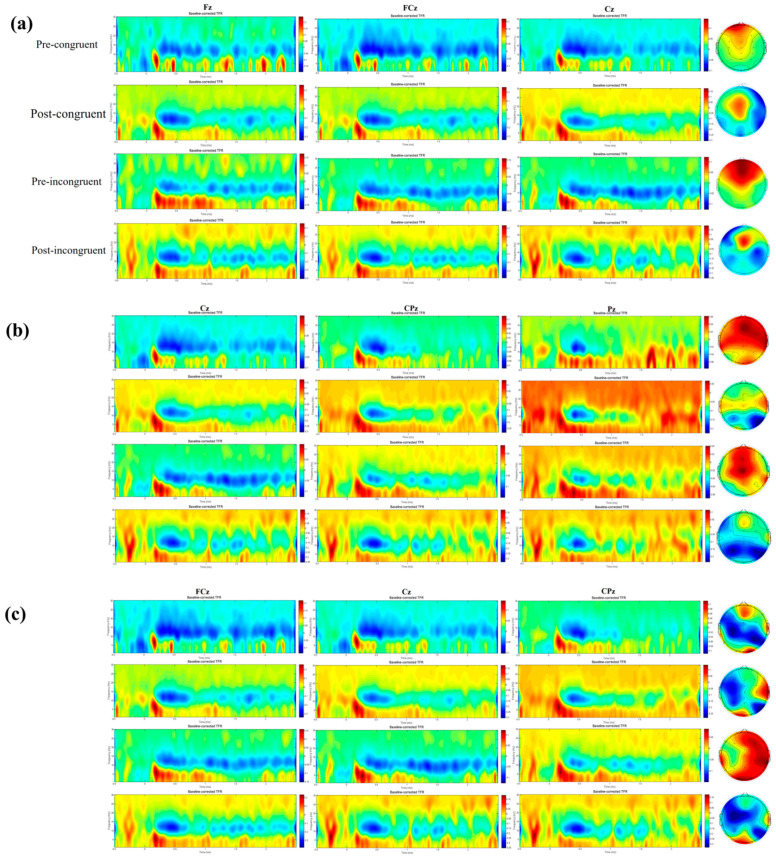
Theta, Alpha and Band Spectral Power Topography. Note: (**a**) Theta-band spectral power topography; (**b**) Alpha-band spectral power topography; (**c**) Beta-band spectral power topography.

A three-way repeated-measures ANOVA with factors of 2 (Time: pre-exercise, post-exercise) × 2 (Congruency: congruent, incongruent) × 3 (Electrode: Fz, FCz, Cz) was performed on the ERSP values in the theta frequency band. The results (Table 4) revealed a significant main effect of Electrode (F(1.281, 43.568) = 18.892, *p* < 0.001, η^2^ = 0.357). Post hoc comparisons indicated that theta-band neural oscillatory activity differed across brain regions, with the highest power at the central site (Cz), which was significantly greater than that at the frontal (Fz) and fronto-central (FCz) sites. However, the main effects of Time and Congruency, as well as all two-way and the three-way interactions, were not significant (*p* > 0.05). The spectral power topography and topographic map of Theta are shown in Figure 3a.

#### 3.4.2. Alpha Band Analysis Results

A three-way repeated-measures ANOVA with factors of 2 (Time: pre-movement, post-movement) × 2 (Congruency: congruent, incongruent) × 3 (Electrode: Cz, Pz, CPz) was performed on the ERSP values in the alpha frequency band (Table 4 and Table S4). The results revealed a significant main effect of Time (F(1, 34) = 11.126, *p* = 0.002, η^2^ = 0.247), indicating a significant decrease in alpha-band power after movement compared to pre-movement. The main effect of Electrode was also significant (F(1.380, 46.932) = 5.474, *p* = 0.015, η^2^ = 0.139). Post hoc comparisons showed that the alpha power at the parietal site (Pz) was significantly higher than that at the central (Cz) and centro-parietal (CPz) sites. However, the main effect of Congruency was not significant. Furthermore, none of the interaction effects reached significance (*p* > 0.05). Figure 3b shows the spectral power topography and topographic map of Alpha.

#### 3.4.3. Beta Band Analysis Results

A three-way repeated-measures ANOVA conducted on the ERSP values in the beta frequency band with factors of Time (pre-movement, post-movement), Congruency (congruent, incongruent), and Electrode (FCz, CPz, Cz) revealed no significant main effects or interaction effects (*p* > 0.05). The spectral power topography and topographic map of Beta are illustrated in Figure 3c Spectral power values for the different frequency bands are provided in Appendix C.

## 4. Discussion

The present study investigated the neurophysiological mechanisms underlying the impairment of cognitive control following acute exercise-induced fatigue. By combining behavioral measures with event-related potential (ERP) and event-related spectral perturbation (ERSP) analyses during a Stroop task, we provide a multi-level account of how physical exhaustion impacts executive function. Our principal findings reveal that acute fatigue led to a significant decline in accuracy specifically during high-conflict (incongruent) trials. This behavioral deficit was mirrored by distinct neural modulations: a reduction in the frontal P3 amplitude for incongruent trials and a global decrease in post-stimulus alpha band power after exercise. Conversely, earlier conflict detection processes, as indexed by the N2 component and theta band oscillations, appeared largely unaffected. These results collectively suggest that acute physical fatigue does not impair the initial detection of cognitive conflict but rather compromises the subsequent allocation of attentional resources required for its resolution, operating within a less efficient cortical environment.

### 4.1. Impairment of Cognitive Control Performance by Acute Exhaustive Exercise

This study found that acute physical exhaustion significantly decreased the participants’ accuracy in the Stroop task under the word-color incongruent (high-conflict) condition, while reaction time did not show significant changes. This result supports our initial hypothesis that exhaustion-inducing exercise impairs cognitive control functions, especially inhibitory control and conflict resolution abilities. In high-conflict tasks, individuals need to mobilize more cognitive resources to inhibit automatic reading responses and focus on reporting the font color as the main task [51]. The decrease in accuracy after exhaustion indicates a weakening of this inhibitory ability. This phenomenon is consistent with the cognitive resource theory, which suggests that high-intensity physical exertion competes with limited brain processing resources for complex cognitive tasks [52]. When the physiological system is at its limit, the resources available for higher-level cognitive functions (such as executive control) will decrease, leading to a decline in task performance.

However, the response time did not significantly change due to fatigue-induced exercise. This may reflect a “speed-accuracy trade-off” strategy, where participants sacrifice behavioral accuracy in order to maintain response speed. Additionally, this may also indicate that acute fatigue primarily affects the quality of cognitive processing (i.e., the efficiency of conflict resolution) rather than the speed of processing. Ramand found that the impact of fatigue on cognitive function is not always global, but may be specific to certain cognitive subcomponents [53].

### 4.2. Neurophysiological Correlates of Fatigue-Induced Cognitive Decline

The ERP results provide a window into the neural dynamics underlying this behavioral impairment. The most salient finding was the significant three-way interaction among time, congruency, and electrode site for the P3 amplitude. Decomposing this interaction revealed that at the frontal Fz electrode, P3 amplitude was significantly reduced post-exercise, but critically, only during the high-conflict incongruent trials. The P3 component is widely interpreted as reflecting the allocation of attentional resources for stimulus evaluation and context updating in working memory [54]. The frontal distribution of this effect points specifically to the disruption of top-down executive control processes mediated by the prefrontal cortex. Following exhaustive exercise, the brain appears to allocate fewer attentional resources to resolve the conflict inherent in the incongruent Stroop task, resulting in the observed increase in errors.

In contrast to the P3 amplitude, the N2 component, which is associated with the earlier stage of conflict monitoring [55], showed a more complex pattern. While we observed a main effect of congruency at the Cz electrode, consistent with the N2’s role in detecting conflict, the significant three-way interaction suggests that the processing of this conflict signal was altered by fatigue in a location-dependent manner. The persistence of a conflict-related N2 signal, followed by a diminished conflict-related P3 signal, suggests a specific breakdown in the cognitive control cascade. The brain successfully detects the presence of conflict (N2) but fails to subsequently mobilize the necessary attentional resources to effectively resolve it (P3), leading to performance decrements.

The P3 latency results add another layer of complexity. The finding that P3 latency was shorter post-exercise for simple, congruent trials at the Pz site is counterintuitive but may reflect the influence of physiological arousal. Intense exercise elevates arousal, which can facilitate processing speed for simple, well-rehearsed tasks. However, this facilitatory effect appears to be overwhelmed by the negative impact of resource depletion when faced with a more complex task requiring conflict resolution. This highlights that the impact of exercise on cognition is not monolithic; it can simultaneously facilitate simple processes through arousal while impairing complex ones through fatigue.

### 4.3. Disruption of Inhibitory Control-Related Neural Oscillations

Our oscillatory data reveal a specific neurophysiological signature of fatigue. The most prominent finding was a significant decrease in alpha-band power over parietal regions following exhaustive exercise. This enhanced alpha event-related desynchronization (event-related desynchronization, ERD) presents a paradox. While alpha ERD is typically interpreted as a marker of active cortical engagement, its occurrence alongside behavioral impairment suggests it may not reflect efficient processing in this context. Instead, we propose it signifies a state of neural inefficiency or a breakdown of inhibitory control. Given that alpha oscillations are thought to gate information flow by inhibiting task-irrelevant neural activity [56] a pronounced decrease in alpha power may indicate a failure to suppress distracting information. This would result in a “noisier” cognitive environment, necessitating more effortful but less effective processing—an interpretation consistent with load theory [57]. Consequently, the fatigued brain may enter a state of hyper-activation or inefficient engagement, expending more effort for a diminished behavioral outcome.

Equally critical to this narrative is the absence of a significant change in midfrontal theta power. Midfrontal theta oscillations are a robust neural signature of cognitive control demand, reflecting conflict detection processes that originate in the medial frontal cortex, including the anterior cingulate cortex [58,59]. The preservation of theta power despite clear behavioral impairment indicates that the initial “alarm signal” for cognitive conflict remains intact during fatigue. The brain continues to detect the need for additional control in challenging situations, such as incongruent Stroop trials. This key finding enables a clear dissociation between the monitoring and implementation stages of cognitive control [60]. It appears that acute exhaustive fatigue spares the conflict monitoring system itself but compromises the subsequent recruitment and implementation of control, processes largely attributed to the lateral prefrontal cortex [61,62].

When integrated, these oscillatory findings present a coherent model of fatigue-related cognitive impairment: the brain detects the need for control (stable theta) but fails to execute it effectively, leading to inefficient cortical processing and a deficient inhibition of irrelevant information (increased alpha ERD). This pattern suggests a functional disconnection or a decline in communication efficiency between the medial frontal monitoring regions and the lateral frontoparietal networks responsible for control implementation [63]. This mechanism carries significant theoretical weight, as it supports multi-stage models of cognitive control and demonstrates how a global physiological state like fatigue can selectively target specific neural computations. Furthermore, these findings resonate with clinical conditions marked by severe fatigue and cognitive dysfunction, such as Myalgic Encephalomyelitis/Chronic Fatigue Syndrome (ME/CFS) [23,64] and Post-COVID-19 Syndrome (PCS) [65,66], where post-exertional malaise is a defining feature. Our acute fatigue model may thus offer insights into the chronic neural dynamics disrupted in these patient populations.

The practical implications of this dissociation are substantial. In high-stakes professions—from athletics and military operations to emergency services and aviation—personnel are often required to make critical decisions under extreme physical exhaustion. Our results indicate that while these individuals may still be able to detect a problem, their capacity to execute the appropriate corrective action is likely impaired. This underscores the critical need for strategies to mitigate fatigue effects, such as implementing structured rest periods, exploring nutritional interventions [67], or developing neuro-monitoring tools that can alert individuals to states of compromised cognitive control.

Furthermore, the null finding regarding beta-band oscillations further refines our model. Beta oscillations are implicated not only in motor preparation but also in maintaining the current cognitive set and stabilizing higher-order cortical networks [68]. The stability of beta activity under fatigue suggests that acute exhaustive exercise does not disrupt the brain’s macroscopic ability to maintain task goals. This reinforces the notion of a selective impairment: fatigue does not induce a global neural collapse but specifically targets processes related to dynamic attentional allocation (alpha) and response implementation, while preserving both conflict monitoring (theta) and task-set maintenance (beta) [69]. This “function-specific impairment” pattern provides a more precise and nuanced understanding of the neural mechanisms underlying fatigue.

### 4.4. Significance and Practical Implications

The findings of this study hold significant theoretical and practical implications. Theoretically, the results provide further support for the neurocognitive model that conceptualizes fatigue as a state of depleted cognitive resources. Both the physical fatigue induced in this study and the mental fatigue elicited by prolonged cognitive tasks in the previous literature demonstrate impairing effects on executive function [70], suggesting they may share underlying psychophysiological mechanisms. The prefrontal cortex, a key region for cognitive control, has high metabolic demands and is particularly vulnerable to systemic physiological changes, such as hypoglycemia or hypoxia, that can accompany exhaustive exercise. The observed reduction in P3 amplitude likely reflects a neurophysiological state of prefrontal hypoactivation, indicating a diminished capacity to engage top-down control circuits.

On a practical level, these findings offer valuable insights for managing and mitigating fatigue. The recently developed “Brain Endurance Training” has been shown to enhance an individual’s resilience to fatigue and improve performance stability under fatigued conditions [71,72]. This indicates that the “resource limitations” observed in our study are not fixed but can be ameliorated through targeted training. Furthermore, various non-pharmacological interventions, such as moderate exercise, yoga, and cognitive behavioral therapy, have demonstrated efficacy in managing fatigue in clinical populations including multiple sclerosis [73], cancer survivors [74], and fibromyalgia [75]. Collectively, this evidence reveals a dose-dependent relationship between exercise and cognitive function: acute exhaustive exercise can be detrimental, whereas chronic moderate exercise has protective and enhancing effects.

Moreover, the results of this study have direct relevance for numerous high-risk professions. In fields such as competitive sports, military operations, emergency response, and aviation, personnel are often required to make critical decisions while experiencing significant physical fatigue. Our findings indicate that even though individuals may still detect conflicts or risks under fatigue (as indexed by the preserved N2 component), their ability to subsequently implement effective corrective actions (reflected by the attenuated P3 component) can be substantially compromised. Therefore, it is crucial to develop and implement effective anti-fatigue strategies. These could include structured work-rest cycles, nutritional interventions [67], or even neurofeedback devices capable of monitoring prefrontal cortex status in real-time to provide early warnings before significant declines in cognitive control occur, thereby safeguarding operational safety and performance stability in high-stakes environments.

### 4.5. Study Limitations

This study has several limitations that warrant consideration. Firstly, the exclusive use of a homogeneous sample of healthy, young, right-handed male university students enhances internal validity but restricts the generalizability of our findings to other populations, such as females, older adults, or individuals with different fitness levels. Secondly, the assessment of fatigue relied primarily on heart rate and RPE; while these are valid measures, they do not directly quantify central nervous system fatigue, and future research could benefit from incorporating biochemical markers (e.g., blood lactate, cortisol) or more direct neuroimaging techniques. Thirdly, the cognitive assessment was confined to the Stroop task, a gold-standard measure of inhibitory control; employing a broader battery of tasks (e.g., N-back for working memory, task-switching paradigms) would provide a more comprehensive understanding of how exhaustive exercise impacts the multifaceted construct of executive function. Fourthly, the present report is confined to time-domain ERPs, and future analyses incorporating time–frequency analysis (e.g., event-related spectral perturbation) are needed to elucidate the oscillatory dynamics underlying the observed cognitive effects. Finally, the incremental test to exhaustion represents a specific model of acute, high-intensity fatigue, and the cognitive consequences of other forms of physical exertion, such as prolonged submaximal exercise, may differ and warrant separate investigation.

## 5. Conclusions

This study provides a multi-faceted neurophysiological account of how acute exhaustive exercise impairs cognitive control. Our findings demonstrate a clear dissociation: while the initial detection of cognitive conflict, indexed by the N2 ERP component and mid-frontal theta oscillations, remains robust in a fatigued state, the subsequent implementation of control is significantly compromised. This impairment is characterized by a reduction in frontal P3 amplitude, reflecting a failure to allocate necessary attentional resources for conflict resolution, and a global decrease in alpha power, suggesting a “noisier” and less efficient cortical processing environment. These results support a multi-stage model of cognitive control and reveal that the implementation phase is particularly vulnerable to the effects of physiological exhaustion. This selective deficit has profound implications for performance in demanding real-world settings and provides a potential neurophysiological framework for understanding cognitive symptoms in clinical disorders associated with chronic fatigue.

## Figures and Tables

**Table 1:** Baseline Characteristics of the Participants (*n* = 35).

Characteristic	Value
Age (years)	23.5 ± 2.31
Height (cm)	178.29 ± 6.87
Weight (kg)	76.36 ± 10.73
Body Mass Index (kg/m^2^)	23.89 ± 3.14
Physical Activity Level (MET-min/week)	4034.73 ± 3747.92

**Table 2:** Evaluation Metrics of the Exhaustion Model (*n* = 35).

Evaluation Metric	At Exhaustion
Maximum Heart Rate during Exercise (beats/min)	183.943 ± 9.374
RPE Score (6–20)	19.143 ± 0.355
Time to Exhaustion (min)	13.954 ± 3.201

**Table 3:** Main and Interaction Effects of Time, Congruency and electrode on P3,N2 Mean Amplitude and Peak Latency.

Components	Metric	Source	*F (df)*	*p*	ղ^2^
P3	Mean Amplitude	T Main Effect	1.287(1, 34)	0.265	0.036
		C Main Effect	4.581(1, 34)	0.040	0.119
		E Main Effect	12.107(1.688, 57.386)	<0.001	0.263
		T × C Interaction	0.209(1, 34)	0.650	0.006
		T × E Interaction	2.620(2.115, 71.898)	0.077	0.072
		C × E Interaction	1.529(2.090, 71.065)	0.223	0.043
		T × C × E Interaction	7.307(1.800, 61.195)	0.002	0.177
	Peak Latency	T Main Effect	0.189(1, 34)	0.667	0.006
		C Main Effect	0.317(1, 34)	0.577	0.009
		E Main Effect	13.07(2.909, 98.918)	<0.001	0.278
		T × C Interaction	0.803(1, 34)	0.377	0.023
		T × E Interaction	5.208(2.656, 90.304)	0.003	0.133
		C × E Interaction	1.143(2.184, 74.249)	0.328	0.033
		T × C × E Interaction	13.17(2.684, 91.241)	<0.001	0.279
N2	Mean Amplitude	T Main Effect	9.933(1, 34)	0.003	0.226
		C Main Effect	5.989(1, 34)	0.020	0.150
		E Main Effect	2.408(1.784, 60.642)	0.104	0.066
		T × C Interaction	0.185(1, 34)	0.670	0.005
		T × E Interaction	1.121(2.182, 74.181)	0.335	0.032
		C × E Interaction	2.036(3.163, 107.531)	0.110	0.056
		T × C × E Interaction	2.006(2.614, 88.860)	0.127	0.056
	Peak Latency	T Main Effect	0.084(1, 34)	0.774	0.002
		C Main Effect	1.122(1, 34)	0.297	0.032
		E Main Effect	20.054(2.374, 80.728)	<0.001	0.371
		T × C Interaction	1.223(1, 34)	0.277	0.035
		T × E Interaction	6.299(3.285, 111,704)	<0.001	0.156
		C × E Interaction	0.296(2.876, 97.783)	0.820	0.009
		T × C × E Interaction	16.872(2.622, 89.156)	<0.001	0.332

Note: T: Time; C: Congruency; E: Electrode.

**Table 4:** Main and Interaction Effects of Time and Congruency on Spectral Power.

Frequency Band	Source	*F (df)*	*p*	ղ^2^
Theta	T Main Effect	0.621(1, 34)	0.436	0.018
	C Main Effect	1.514(1, 34)	0.227	0.043
	E Main Effect	18.892(1.281, 43.568)	<0.001	0.357
	T × C Interaction	2.320(1, 34)	0.137	0.064
	T × E Interaction	0.182(1.162, 39.491)	0.709	0.005
	C × E Interaction	1.512(1.288, 43.797)	0.230	0.043
	T × C × E Interaction	0.113(1.302, 44.274)	0.803	0.003
Alpha	T Main Effect	11.126(1, 34)	0.002	0.247
	C Main Effect	0.235(1, 34)	0.631	0.007
	E Main Effect	5.474(1.380, 46.932)	0.015	0.139
	T × C Interaction	1.484(1, 34)	0.232	0.042
	T × E Interaction	0.062(1.242, 42.226)	0.855	0.002
	C × E Interaction	0.187(1.604, 54.530)	0.781	0.005
	T × C × E Interaction	0.315(1.435, 48.787)	0.659	0.009
Beta	T Main Effect	1.804(1, 34)	0.188	0.050
	C Main Effect	0.217(1, 34)	0.644	0.006
	E Main Effect	0.840(1.189, 40.425)	0.384	0.024
	T × C Interaction	2.532(1, 34)	0.121	0.069
	T × E Interaction	1.130(1.141, 38.807)	0.303	0.032
	C × E Interaction	2.232(1.337, 45.456)	0.135	0.062
	T × C × E Interaction	0.165(1.193, 40.552)	0.731	0.005

## Data Availability

The original contributions presented in this study are included in the article/Supplementary Material. Further inquiries can be directed to the corresponding author.

## Supplementary Materials

The following supporting information can be downloaded at: https://www.mdpi.com/article/10.3390/biology14121688/s1, Table S1: The maximum heart rate, time to exhaustion, and subjective rating of perceived exertion were recorded for each subject during the exhaustive exercise protocol. Table S2: The behavioral data of subjects during the Stroop task under four conditions: Pre-congruent, Post-congruent, Pre-incongruent, and Post-incongruent, including accuracy rate and reaction time. Table S3: P3 and N2 component metrics across experimental conditions and electrode sites. Provides detailed values of P3 and N2 component characteristics including mean amplitude and peak latency recorded from five electrode positions (Fz, FCz, Cz, CPz, Pz) under congruent and incongruent conditions, before and after exercise. Table S4: Neural Oscillatory Activity in Frequency Bands. Displays spectral power values of neural oscillations in theta, alpha, and beta frequency bands recorded from selected elec-trode sites under congruent and incongruent conditions, before and after exercise.

## Abbreviations

The following abbreviations are used in this manuscript:

EEG	Electroencephalogram
ERP	Event-Related Potential
ERSP	Event-Related Spectral Perturbation
ACC	Anterior Cingulate Cortex
RPE	Rating of Perceived Exertion
ANOVA	Analysis of Variance
ERD	Event-Related Desynchronization

## Appendix A

**Table A1:** P3 component metrics across experimental conditions and electrode sites.

Metric	Electrode	Congruent		Incongruent	
		Pre	Post	Pre	Post
Mean Amplitude (μV)	Fz	0.823 ± 1.841	0.429 ± 2.289	0.581 ± 1.801	−0.256 ± 1.987
	FCz	0.964 ± 1.844	0.734 ± 2.211	0.602 ± 1.459	0.158 ± 1.969
	Cz	1.308 ± 1.506	1.058 ± 1.868	0.681 ± 1.394	0.709 ± 2.252
	CPz	1.604 ± 1.511	1.327 ± 1.677	1.187 ± 1.498	1.325 ± 2.253
	Pz	1.375 ± 1.304	0.963 ± 1.610	1.039 ± 1.285	1.420 ± 2.128
Peak Latency (ms)	Fz	353.247 ± 26.432	361.272 ± 18.282	357.701 ± 24.729	355.580 ± 20.939
	FCz	351.118 ± 27.325	357.031 ± 20.213	355.804 ± 26.734	354.464 ± 22.145
	Cz	349.554 ± 27.203	356.361 ± 19.509	352.567 ± 27.251	353.013 ± 22.038
	CPz	350.893 ± 23.332	347.210 ± 25.941	356.138 ± 21.152	348.326 ± 23.958
	Pz	347.176 ± 23.168	335.272 ± 24.813	347.594 ± 22.623	341.183 ± 24.190

## Appendix B

**Table A2:** N2 component metrics across experimental conditions and electrode sites.

Metric	Electrode	Congruent		Incongruent	
		Pre	Post	Pre	Post
Mean Amplitude (μV)	Fz	−0.827 ± 2.809	−1.454 ± 2.752	−0.783 ± 1.746	−2.200 ± 2.342
	FCz	−0.613 ± 2.580	−1.257 ± 2.595	−0.680 ± 1.623	−1.904 ± 2.368
	Cz	−0.236 ± 1.978	−1.056 ± 2.367	−0.782 ± 1.788	−1.359 ± 2.122
	CPz	−0.291 ± 1.752	−0.780 ± 2.078	−0.506 ± 1.551	−0.825 ± 2.129
	Pz	−0.227 ± 1.125	−0.755 ± 1.536	−0.533 ± 1.233	−0.718 ± 1.167
Peak Latency (ms)	Fz	252.549 ± 36.880	266.226 ± 27.004	261.212 ± 28.333	262.449 ± 35.322
	FCz	249.690 ± 38.361	260.465 ± 27.975	261.708 ± 24.727	259.995 ± 35.062
	Cz	246.407 ± 35.953	243.299 ± 31.863	259.362 ± 26.994	251.749 ± 36.456
	CPz	243.502 ± 31.542	234.463 ± 36.753	248.105 ± 31.484	241.615 ± 35.763
	Pz	230.463 ± 37.279	227.344 ± 39.563	230.346 ± 36.643	219.420 ± 36.589

## Appendix C

**Table A3:** Neural Oscillatory Activity in Frequency Bands.

Metric	Electrode	Congruent (μV)		Incongruent (μV)	
		Pre	Post	Pre	Post
θ	Fz	0.134 ± 1.328	0.436 ± 1.268	0.529 ± 1.092	0.532 ± 1.067
	FCz	0.287 ± 1.219	0.611 ± 1.203	0.646 ± 1.084	0.627 ± 1.100
	Cz	0.439 ± 1.081	0.726 ± 1.121	0.771 ± 1.072	0.707 ± 1.035
α	Cz	−0.587 ± 1.123	−0.775 ± 1.164	−0.342 ± 1.097	−0.907 ± 1.154
	CPz	−0.653 ± 1.164	−0.946 ± 1.229	−0.505 ± 1.142	−1.038 ± 1.211
	Pz	−0.790 ± 1.308	−1.079 ± 1.318	−0.599 ± 1.484	−1.125 ± 1.158
β	FCz	−0.901 ± 1.032	−0.893 ± 0.815	−0.807 ± 1.233	−1.215 ± 1.155
	Cz	−0.891 ± 0.918	−0.849 ± 0.784	−0.733 ± 0.899	−1.159 ± 1.163
	CPz	−0.978 ± 0.974	−0.875 ± 0.716	−0.741 ± 0.834	−1.015 ± 1.181
